# Seasonal variation in space use and territoriality in a large mammal (*Sus scrofa*)

**DOI:** 10.1038/s41598-022-07297-y

**Published:** 2022-03-07

**Authors:** Peter E. Schlichting, Raoul K. Boughton, Wes Anderson, Bethany Wight, Kurt C. VerCauteren, Ryan S. Miller, Jesse S. Lewis

**Affiliations:** 1 College of Integrative Sciences and Arts, Arizona State University, Polytechnic Campus, 6073 South Backus Mall, Mesa, AZ 85212 USA; 2 University of Florida, Range Cattle Research and Education Center, Wildlife Ecology and Conservation, 3401 Experiment Station, Ona, FL 33865 USA; 3 United States Department of Agriculture, Animal and Plant Health Inspection Service, Wildlife Services, National Wildlife Research Center, 4101 LaPorte Avenue, Fort Collins, CO 80521-2154 USA; 4 United States Department of Agriculture, Animal and Plant Health Inspection Service, Veterinary Services, Center for Epidemiology and Animal Health, 2150B Center Avenue, Fort Collins, CO 80526 USA; 5 Present Address: Illinois Department of Natural Resources, 1 Natural Resources Way, Springfield, IL 62702 USA

**Keywords:** Ecology, Zoology, Ecology

## Abstract

An individual’s spatial behavior is shaped by social and environmental factors and provides critical information about population processes to inform conservation and management actions. Heterogeneity in spatial overlap among conspecifics can be evaluated using estimates of home ranges and core areas and used to understand factors influencing space use and territoriality. To understand and test predictions about spatial behavior in an invasive large mammal, the wild pig (*Sus scrofa*), we examined variation in space use between sexes and seasons. We predicted that if animals were territorial that there would be a reduction in space-use overlap when comparing overlap of home ranges (HR–HR), to home ranges and core areas (HR–CA), and in-turn between core areas (CA–CA). Home ranges and core areas were estimated for 54 wild pigs at Buck Island Ranch, FL from GPS telemetry data. Overlap indices were calculated to estimate the strength (space-use overlap) and number of potential interactions within three wet seasons (June–October) and two dry seasons (December–April). Among sexes, home range size did not vary seasonally, and males exhibited larger home ranges compared to females (M = 10.36 ± 0.79 km^2^ (± SE), F = 3.21 ± 0.16 km^2^). Strength of overlap varied by season with wild pig home ranges overlapping more during the dry season. Males interacted with a greater number of individuals of both sexes, compared to females, and exhibited greater strength of overlap during the dry season. Consistent with our predictions, wild pigs appeared to exhibit territorial behavior, where strength of overlap decreased when comparing HR–HR to HR–CA and HR–CA to CA–CA. Our framework can be used to understand patterns of space use and territoriality in populations, which has important implications in understanding intraspecific interactions and population processes, such as how pathogens and parasites might spread within and among populations.

## Introduction

Variation in spatial interactions among animals can identify the selective pressures that shape spatial behavior. The relationship between the spatial organization of a species and dynamic interactions among individuals is expected to vary based on both behavioral and environmental factors. Differences between males and females in spatial behavior are related to differing reproductive strategies and spatial requirements allometrically scaling with body size^1–3^, which can influence home range size^4^ and habitat use^5^. Seasonal changes in behavioral and environmental influences can also modify spatial behavior and influence overlap and association of conspecifics^4,6–8^. Spatial overlap of individuals influences intraspecific interactions, which can drive population processes, such as disease transmission^9^, survival^10,11^, competition^12^, and reproduction^13^. Despite the growing evidence that spatial relationships affect a diverse range of socio-ecological processes^14^, variation in spatial overlap patterns between sexes and seasons is poorly understood for many species and is dependent on the individual’s spatial organization. Identifying factors that influence heterogeneity in spatial overlap can improve management and conservation decisions by understanding important time periods for disease transmission^15^, intraspecific competition^12^, and management actions^16^.

**Figure 1:**
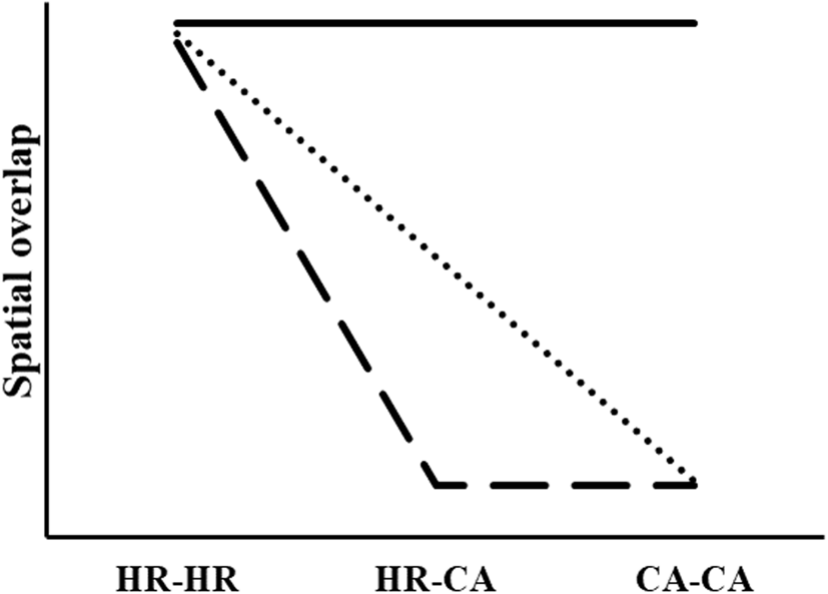
Predicted relationships evaluating territoriality among animals based on spatial overlap in home ranges (HR) and core areas (CA). Interactions can occur between two individual’s home ranges (HR–HR), between one individual’s home range and another individual’s core area (HR–CA), or between two individual’s core areas (CA–CA). If animals are not territorial, spatial overlap is expected to be the same across HR–HR, HR–CA, and CA–CA comparisons (solid line). If animals are territorial, it is expected that spatial overlap will decrease between HR–HR and HR–CA comparisons [dashed line; consistent with Burt (1943) and Schoener (1968)] or among HR–HR, HR–CA, and CA–CA comparisons [dotted line; consistent with Gabor et al. (1999), Darden and Dabelsteen (200), and Pierro et al. (2008)].

**Figure 2:**
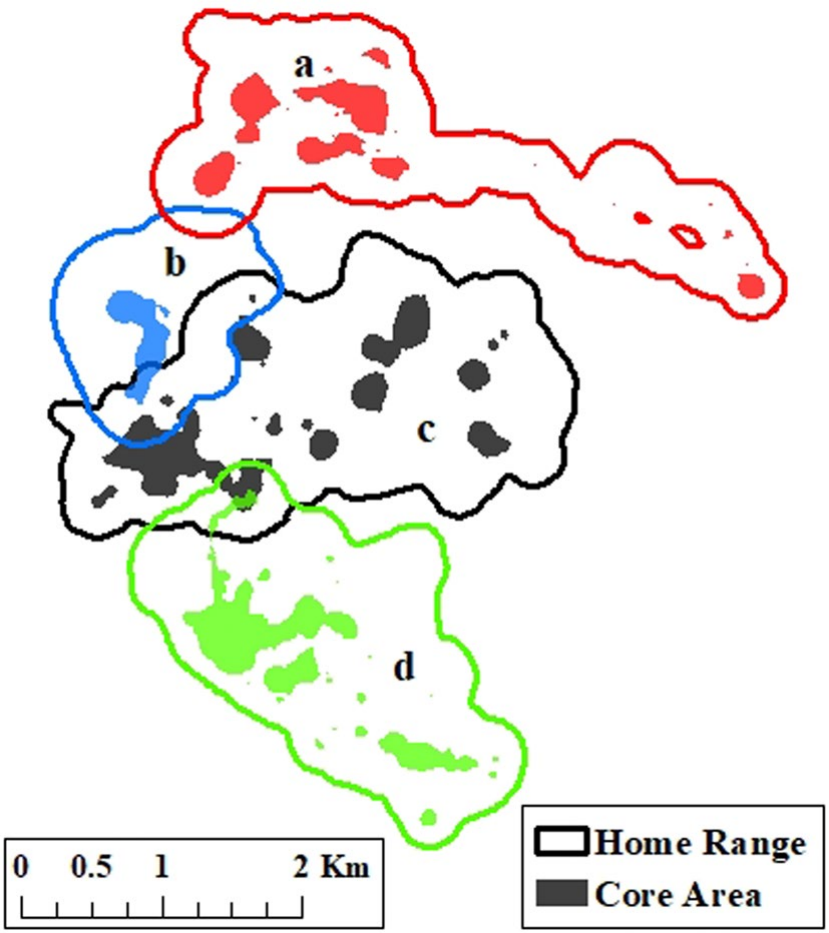
Home range (HR) and core area (CA) overlap of 4 female wild pigs at Buck Island Ranch, FL, during the Dry 2017 season. As an example, overlap of home ranges (HR–HR) occurs between 3 pairs of individuals (a–b, b–c, and c–d), overlap of home ranges and core areas (HR–CA) occurred between two pairs (b–c and c–d), and core area overlap (CA–CA) occurred between one pair (c–d). Figure was created in ArcMap 10.1.

Spatial behavior through time can be characterized by an individual’s home range and territory. Home ranges are selected by animals to maximize their survival and reproduction, with territories traditionally defined as a reduced portion of the home range that is actively defended or exclusively used^17–20^. Territorial behavior can increase an individual’s fitness by excluding conspecifics from areas containing limiting resources, including food, cover, and mates. There is considerable variation, however, in how territories are defined and interpreted^19,20^ and they are often approximated by estimating core areas (i.e., areas of high probability of use within an animal’s home range^21–23^. Although the identification of core areas can be subjective, territoriality can be identified by examining overlap between adjacent individual’s home range and core areas^24–26^. Non-territorial species are expected to overlap with conspecifics at random which would result in overlap with a similar number of individuals in home ranges and core areas (Fig. 1). Conversely, territorial behavior creates spatial heterogeneity in the number and strength of conspecific interactions, leading to reduced overlap of core areas compared to home ranges (Fig. 1). In the paradigm proposed by Burt^17^ and Schoener^18^, territorial species overlap in their home ranges but have exclusive core areas and thus home ranges do not overlap core areas between neighboring individuals (Figs. 1, 2). Other studies have defined territoriality as occurring when core areas are mutually exclusive between animals, but home ranges overlap with core areas of adjacent individuals^23,24,26–28^ (Figs. 1, 2). It is predicted that if animals are territorial that there will be a reduction in spatial overlap when comparing overlap of home ranges (HR–HR), home ranges and core areas (HR–CA), and core areas (CA–CA) (Fig. 1). If animals are not territorial and spatial overlap among individuals is random, then it would be predicted that overlap would be similar when comparing HR–HR, HR–CA, and CA–CA (Fig. 1). Spatial overlap patterns could be used to quantify territoriality in many species and across a range of different social structures, but this approach could be particularly useful for species that exhibit heterogeneity in space use yet lack conspicuous territorial behaviors or exclusive core areas.

Wild pigs, *Sus scrofa*, are a large-bodied, gregarious species with an expansive native and invasive range, ecological role as an ecosystem engineer, and complex socio-spatial behavior^29–32^. Seasonal wild pig home ranges can vary inversely with forage availability and other landscape features^33–40^, which could influence spatial overlap^41,42^ and territorial patterns^12,42,43^. Although territorial patterns have been predicted for wild pigs^44^, previous studies have provided conflicting evidence. Female groups are argued to be territorial with other female groups where groups overlap in their home ranges, but exhibit mutually exclusive core areas^27,45,46^. This pattern, however, is not detected in other systems^35,47,48^ and the role of territoriality in wild pig social structure is unclear. Males are reported to be less territorial than females (but see Ref.^35^), with males exhibiting larger home ranges that overlap with both sexes^49^. How home range size, spatial overlap, and territoriality vary across seasons has not been widely evaluated for wild pigs and it is unclear how different behavioral and environmental factors influence space-use overlap.

Here we examined variation in the spatial behavior of wild pigs and used this information to understand patterns of territoriality. Our first objective was to examine variation in home range size and spatial overlap between sexes and seasons. We expected that males would (1) exhibit larger home ranges, (2) exhibit increased space-use overlap compared to females^49,50^ and (3) interact with a greater number of conspecifics within their home ranges. Our second objective was to evaluate how home range size and space-use overlap varied seasonally. Assuming forage availability to be lower during the dry season than the wet season^51,52^, we predicted that home range size and space-use overlap would be greater during dry seasons because animals would use larger areas to acquire sufficient resources^38^. Conversely, if resources are more concentrated and limiting during the dry season, then wild pigs might exhibit less space-use overlap during this season. Our third objective was to use measures of spatial overlap to evaluate territoriality of home ranges and core areas and how overlap varied by sex and season. If wild pigs exhibited territoriality, we expected that spatial overlap among individuals would be greatest for home range to home range (HR–HR) interactions, reduced for home range to core area (HR–CA) interactions, and lowest for core area to core area (CA–CA) interactions (Fig. 1). We predicted that males and females would differ in their spatial overlap, with females exhibiting more exclusive core areas. Territoriality was expected to be strongest during the dry season as resources are assumed to be more limiting.

## Results

**Figure 3:**
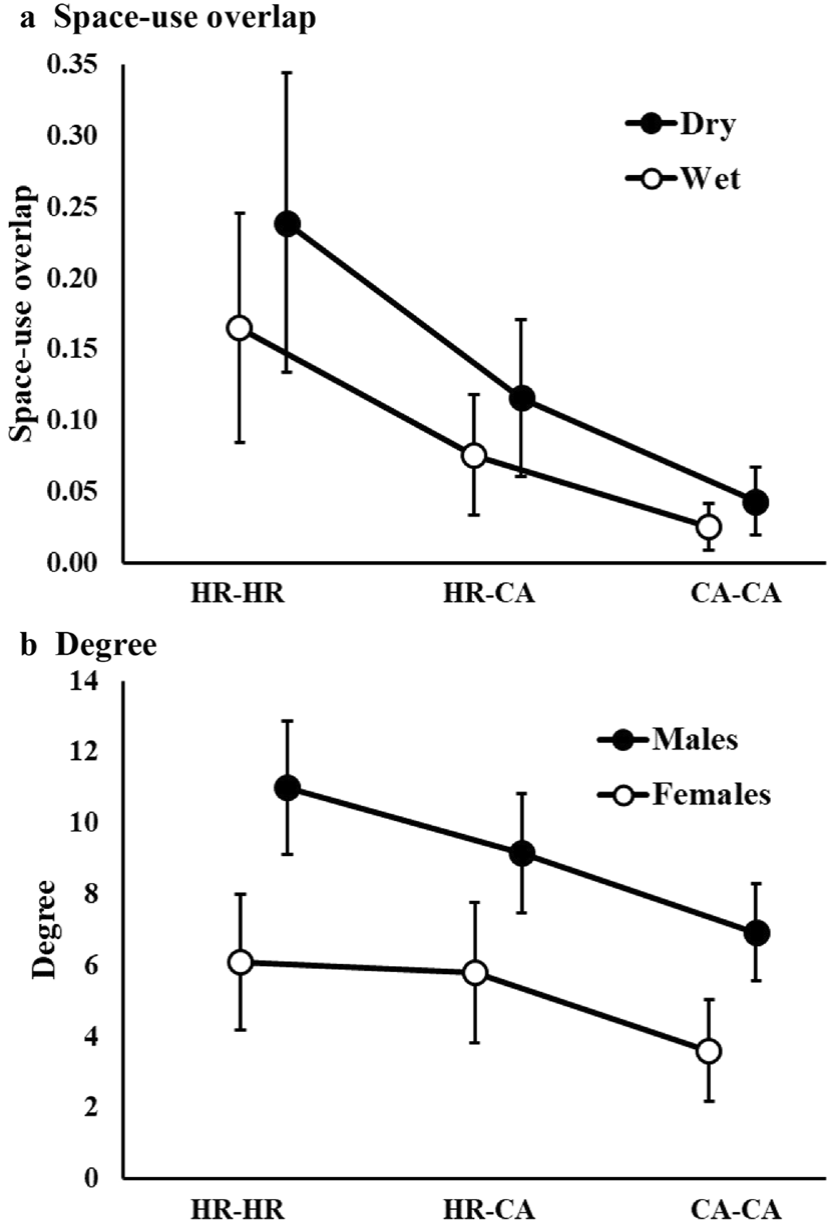
Space-use overlap (measured by utilization distribution of overlap index (UDOI); (**a**) and degree (number of individuals overlapped; (**b**) estimates with associated 95% confidence intervals for wild pigs at Buck Island Ranch, FL. Space-use overlap estimates come from all seasons while degree represents the Dry 2016 season which contained the greatest number of monitored individuals. Estimates were created for home range to home range (HR–HR), home range to core area (HR–CA) and core area to core area (CA–CA) overlap. Space use overlap was estimated for dry (Dry) and wet (Wet) seasons. Degree values were estimated for male (M) and female (F) wild pigs.

Spatial data from 54 individuals was used to create 80 seasonal wild pig home ranges across multiple years, with the number of monitored individuals and sex ratio varying by season and sampling period (Tables 1 and 2). Following predictions in objectives one and two, males and females differed in home range size with females having smaller home ranges than males in wet and dry seasons (Table 1). The highest ranked model for estimating home range size included sex and it accounted for 95% of model weight with no other models within Δ2 AIC (Supplementary Table S1a). For space-use overlap of home ranges, the highest ranked model included the covariates season and sex, with the top two models accounting for 77% of model weight (Supplementary Table S1b). Space-use overlap (Ave. ± SE) was greater during the dry season compared to the wet season (Fig. 3a.) and lowest for interactions among females (Dry = 0.010 ± 0.033, Wet = 0.098 ± 0.025), intermediate for interactions among males (Dry = 0.230 ± 0.041, Wet = 0.174 ± 0.052), and greatest for female-male interactions (Dry = 0.264 ± 0.065, Wet = 0.190 ± 0.048; Supplementary Fig. S1). For degree, the highest ranked model included sex (Supplementary Table S1c), and males overlapped with more individuals in both seasons, compared to females (Table 2, Fig. 3b). Season was also included in a competitive model with greater overlap during the dry season (Supplementary Table S1c). For analyses of both home range size and space-use overlap, including year as a random effect did not improve model fit and it was excluded from all models with fixed effects (Supplementary Table S1).

**Table 1:** Average (Ave.) seasonal home range and core area sizes (km^2^) for wild pigs at Buck Island Ranch, FL. Home range information was provided for all wet and dry seasons. Home range information was divided by sex (F: female, M: male) with sample size (n), standard error (S.E.), minimum values (Min.), and maximum values (Max.) included.

Season	Sex	n	Home range				Core area			
			Ave	S.E	Min	Max	Ave	S.E	Min	Max
Wet	F	25	3.54	0.28	1.26	6.80	0.49	0.05	0.11	0.93
	M	18	10.16	1.26	1.61	22.97	1.27	0.17	0.08	2.81
Dry	F	18	3.59	0.32	1.22	6.67	0.50	0.05	0.17	1.02
	M	19	10.66	1.05	4.70	23.25	1.20	0.13	0.35	2.48

**Table 2:** Seasonal estimates of degree (number of animals an individual overlapped with in space use) for female (F) and male (M) wild pigs at Buck Island Ranch, Florida. We estimated degree among home ranges (HR–HR), between home ranges and the individual’s core area (HR–CA), and among core areas (CA–CA). Degree information includes standard error (S.E.), minimum values (Min.), and maximum values (Max).

	Wet 2015		Dry 2016		Wet 2016		Dry 2017		Wet 2017	
	F	M	F	M	F	M	F	M	F	M
**a. HR–HR**										
Average	5.00	7.17	6.10	11.00	4.73	6.67	3.13	4.83	2.50	3.00
S.E	0.84	0.79	0.97	0.93	0.84	1.15	0.64	1.11	0.38	0.52
Min	2	4	2	5	1	3	1	1	1	1
Max	7	9	12	15	9	10	6	8	4	4
**b. HR–CA**										
Average	4.60	6.17	5.80	9.15	3.27	5.33	2.88	4.83	2.00	2.17
SE	0.93	0.83	1.01	0.99	0.56	1.12	0.67	1.11	0.27	0.60
Min	1	4	2	5	1	1	1	1	1	0
Max	6	9	12	14	6	9	6	8	3	4
**c. CA–CA**										
Average	2.60	4.17	3.60	6.92	2.45	4.17	2.38	4.50	0.75	1.33
SE	0.68	0.83	0.73	0.90	0.31	0.70	0.65	1.20	0.25	0.33
Min	0	1	1	3	1	1	0	0	0	0
Max	4	7	9	14	4	6	5	8	2	2

Wild pigs exhibited territorial behavior, which was consistent with our predictions for objective three (Figs. 1 and 3a). The highest ranked models for space-use overlap of HRHR–HRCA interactions included the variables level (HR–HR, HR–CA), season (Wet or Dry), the interaction between level and season, and sex. The top models accounted for 99% of the model weight (Table 3a). The highest ranked models for HRCA–CACA interactions included the variables level (HR–CA or CA–CA interactions), season (Wet or Dry), and the interaction between level and season. Sex was not included as a covariate in models within Δ2 AIC (Table 3b). Space-use overlap (Ave. ± SE) was greatest for HR–HR interactions (0.207 ± 0.035), intermediate for HR–CA interactions (0.098 ± 0.018) and lowest for CA–CA interactions (0.036 ± 0.008), and overlap was greater in the dry than wet season across home range levels (Fig. 3a). Space-use overlap was lowest for interactions among females, intermediate for male–male interactions, and greatest for female–male interactions. For both HRHR–HRCA and HRCA–CACA analyses of space-use overlap, including year as a random effect did not improve model fit and it was excluded from all models with fixed effects (Table 3a,b).

**Table 3:** Results of generalized mixed-effects linear-regression models evaluating variation in spatial overlap of wild pigs at Buck Island Ranch, FL. Spatial overlap was measured by utilization distribution of overlap index (UDOI, a and b) and degree (c and d). Random effects include the two individuals that are interacting (Dyad) or the individual (ID) and the year (Year) or the sampling period (e.g. Wet 2015, SP). Models include sex (Sex), level (home range-home range (HR-HR), home range–core area (HR–CA), and core area–core area (CA–CA) overlap, and season as fixed effects. For UDOI, sex indicates the sex of the two interacting individuals (two females, female and male, and two males). For degree, sex is the sex of the individual used to determine degree. Model output includes the number of parameters (K), AIC values, ΔAIC values, AIC weights (w_i_), and residual deviance (Dev.).

	K	AIC	ΔAIC	*wi*	Dev
**a. UDOI: HRHR–HRCA**					
(1|Dyad) + Level + Season	5	− 2077.35	–	0.53	1043.71
(1|Dyad) + Level + Season + (Level × Season)	6	− 2075.92	1.43	0.26	1044.01
(1|Dyad) + Level + Sex + Season	7	− 2075.43	1.92	0.20	1044.78
(1|Dyad) + Level + Sex + Season + (Level × Sex × Season)	14	− 2065.31	12.04	0.00	1046.89
(1|Dyad) + Sex + Level	6	− 2064.47	12.88	0.00	1038.28
(1|Dyad) + Level	4	− 2064.25	13.09	0.00	1036.15
(1|Dyad) + Level + Sex + (Level × Sex)	8	− 2060.87	16.48	0.00	1038.52
(1|Dyad) + Season	4	− 2002.05	75.30	0.00	1005.05
(1|Dyad) + Sex + Season	6	− 2000.18	77.16	0.00	1006.14
(1|Dyad) + Sex + Season + (Sex × Season)	8	− 1998.16	79.19	0.00	1007.16
(1|Dyad) + Sex	5	− 1991.35	85.99	0.00	1000.71
(1|Dyad)	3	− 1991.15	86.20	0.00	998.59
(1|Dyad) + (1|Year)	4	− 1989.14	88.21	0.00	998.59
Intercept-only	2	− 1551.06	526.29	0.00	777.54
(1|Year)	3	− 1546.80	530.55	0.00	776.41
**b. UDOI: HRCA–CACA**					
(1|Dyad) + Level + Season	5	− 1289.16	–	0.45	649.65
(1|Dyad) + Level + Season + (Level × Season)	6	− 1287.55	1.61	0.20	649.88
(1|Dyad) + Level	4	− 1286.87	2.29	0.14	647.48
(1|Dyad) + Level + Sex + Season	7	− 1286.61	2.55	0.13	650.44
(1|Dyad) + Level + Sex	6	− 1285.17	3.99	0.06	648.69
(1|Dyad) + Level + Sex + (Level × Sex)	8	− 1283.11	6.05	0.02	649.73
(1|Dyad) + Level + Sex + Season + (Level × Sex × Season)	14	− 1277.25	11.91	0.00	653.15
(1|Dyad) + Season	4	− 1253.95	35.21	0.00	631.02
(1|Dyad)	3	− 1252.29	36.87	0.00	629.17
(1|Dyad) + Sex + Season	6	− 1251.44	37.73	0.00	631.82
(1|Dyad) + Sex	5	− 1250.59	38.57	0.00	630.37
(1|Dyad) + (1|Year)	4	− 1250.25	38.91	0.00	629.17
(1|Dyad) + Sex + Season + (Sex × Season)	8	− 1249.71	39.45	0.00	633.03
Intercept-only	2	− 1092.45	196.71	0.00	548.24
(1|Year)	3	− 1090.42	198.74	0	548.24
**c. Degree: HRHR–HRCA**					
(1|ID) + (1|SP) + Level + Sex	6	695.30	–	0.45	− 341.37
(1|ID) + (1|SP) + Level + Sex + Season	7	696.55	1.25	0.24	− 340.90
(1|ID) + (1|SP) + Level + Sex + (Level × Sex)	7	697.49	2.18	0.15	− 341.37
(1|ID) + (1|SP) + Sex	5	698.97	3.66	0.07	− 344.29
(1|ID) + (1|SP) + Sex + Season	6	700.19	4.89	0.04	− 343.82
(1|ID) + (1|SP) + Sex + Season + (Sex × Season)	7	701.80	6.49	0.02	− 343.52
(1|ID) + (1|SP) + Level	5	702.57	7.26	0.01	− 346.09
(1|ID) + (1|SP) + Level + Season	6	703.69	8.39	0.01	− 345.57
(1|ID) + (1|SP) + Level + Sex + Season + (Level × Sex × Season)	11	704.11	8.81	0.01	− 340.15
(1|ID) + (1|SP) + Level + Season + (Level × Season)	7	705.23	9.93	0.00	− 345.24
(1|ID) + (1|SP)	4	706.26	10.96	0.00	− 349.00
(1|ID) + (1|SP) + Season	5	707.35	12.05	0.00	− 348.48
(1|ID)	3	741.46	46.16	0.00	− 367.65
(1|SP)	3	756.12	60.82	0.00	− 374.98
Intercept-only	2	766.09	70.79	0.00	− 381.01
**d. HRCA–CACA**					
(1ID) + (1|SP) + Level + Sex	6	653.17	–	0.40	− 320.31
(1ID) + (1|SP) + Level + Sex + Season	7	653.47	0.30	0.34	− 319.36
(1ID) + (1|SP) + Level + Sex + (Level × Sex)	7	654.37	1.20	0.22	− 319.81
(1ID) + (1|SP) + Level	5	660.74	7.57	0.01	− 325.17
(1ID) + (1|SP) + Level + Season	6	660.84	7.67	0.01	− 324.14
(1ID) + (1|SP) + Level + Sex + Season + (Level × Sex × Season)	11	660.90	7.73	0.01	− 318.55
(1ID) + (1|SP) + Level + Season + (Level × Season)	7	662.43	9.26	0.00	− 323.84
(1ID) + (1|SP) + Sex + Season	6	669.65	16.48	0.00	− 328.55
(1ID) + (1|SP) + Sex + Season + (Sex × Season)	7	671.63	18.46	0.00	− 328.44
(1ID) + (1|SP)	4	676.96	23.79	0.00	− 334.35
(1ID) + (1|SP) + Season	5	677.04	23.87	0.00	− 333.32
(1ID) + (1|SP) + Sex	4	692.98	39.81	0.00	− 342.36
(1|ID)	3	714.55	61.38	0.00	− 354.20
(1|SP)	3	717.57	64.40	0.00	− 355.71
Intercept-only	2	813.62	160.45	0.00	− 404.77

Further, the metric “degree”, also indicated that wild pigs exhibited territorial behavior. Models that included level, sex of the individual, season, and the interaction of sex and level were the most supported (Table 3c,d). Degree was greatest for HR–HR interactions (5.77 ± 0.24), intermediate for HR–CA interactions (4.89 ± 0.22) and lowest for CA–CA interactions (3.49 ± 0.18; Table 3c,d, Fig. 3b and Supplementary Fig. S2). Males consistently overlapped with a greater number of individuals than females (Table 2, Fig. 3b. and Supplementary Fig. S2). Wild pigs overlapped more conspecifics during the dry than wet season in both analyses and season was included in models within Δ2 AIC (Table 3c,d). The highest ranked models for HRHR–CACA and HRCA–CACA analyses accounted for 84% and 96% of model weight respectively.

## Discussion

Heterogeneity in wild pig spatial behavior identified several factors that shape overlap of individuals and territorial patterns. Consistent with our predictions and previous research, males exhibited greater home range size and interacted with more conspecifics than females likely due to allometric requirements or to maximize reproductive potential^1–3^. Differences in the number of indirect interactions between males and females are likely due to differences in home range size, as individuals with larger home ranges are more likely to overlap with a greater number of neighboring animals^53,54^. This could influence important ecological processes, such as genetic structuring of populations and transmission of pathogens^55,56^. The role of sex in structuring spatial overlap was more nuanced when evaluating the strength of home range overlap. Space-use overlap of female home ranges was consistently lower than female-male and male-male overlap. It was common for male home ranges to completely overlap one or more female home ranges and males overlapped extensively. Female’s expression of territoriality was more robust than males, and the overlap patterns presented here support the hypothesis that females exhibit greater territoriality with neighboring females compared to associated males or between males^50,57^.

The influence of seasonality on wild pig spatial patterns varied depending on how space use was evaluated. Home range size did not vary seasonally in this system. Landscape structure^33,39,58^ and the presence of agriculture^59^ can influence seasonal space use by wild pigs but these factors were not present in this study, potentially limiting seasonal effects. High population density can also influence spatial patterns and potentially limit seasonal variation in home range size^60^, although changes in density have not elicited expansion or shifting of wild pig home ranges in other systems^61^. Although home range size did not vary seasonally, our results suggest that the strength and number interactions were greater during dry seasons. Wild pigs often forage on grasses and below the soil surface by rooting during the dry season, and utilization of these abundant but lower quality resources can mitigate the reductions in productivity^51^. Changes in foraging strategy may result in shifts in space-use within the home range^40^ and a reduction in territoriality^41,62^. Although environmental and social factors likely influenced how seasonal effects were expressed in spatial overlap, territorial patterns were consistently detected from the spatial behavior of wild pigs.

Territoriality exists on a continuum and is influenced by the species’ social structure and ecological conditions^17,19,20^. In our system, core areas were not exclusive, where home ranges of neighboring wild pigs overlapped conspecific core-areas, indicating wild pigs do not conform to some traditional definitions of territoriality^17,18^. Similar to recent work on wild pig territoriality^43^, core areas of neighboring individuals also overlapped based on the 80% cumulative probability isopleth selected for this study. We selected this cumulative probability isopleth to retain a similar proportion of the home range within the core area across individuals. It is challenging to monitor all neighboring social groups and the unknown influence of unmonitored conspecifics, as well as variation in the spatial distribution of monitored individuals, led us to evaluate overlap at predetermined isopleths. Where all neighboring groups are monitored, analysis of overlap at varying isopleth levels could identify a threshold where core area exclusivity is consistently expressed. Exclusive use of areas is likely maintained by indirect communication^27,63,64^ and limited direct interactions^43,50,57,65^. Interactions within shared space may also be limited due to temporal partitioning via avoidance, site specific dominance, or priority of access to resources^19^. Evaluating how wild pigs interact in both time and space, particularly under contrasting resource availability could identify how spatial behavior varies with ecological conditions.

The conceptual framework that we presented (Fig. 1) suggests that spatial overlap patterns can be used to evaluate territoriality in species without overt territorial behavior and non-exclusive core areas. However, there are several considerations about our study that suggest further work is needed to understand the role of territoriality in wild pig social structure. The presence of territoriality within wild pig populations suggests that the sharing of core areas between neighbors is limited and they may contain important and/or limiting resources^62^. Comparing habitat characteristics in core areas and shared home range space could identify limiting resources and improve our understanding of how resources shape territorial patterns in wild pigs^19,43^. Further, spatial interactions and territorial patterns could be evaluated in relation to important resources, such as mast producing trees, supplemental feeders, or at water locations where sharing of space likely occurs. Evaluation of how shared space is segregated temporally would identify the mechanisms maintaining territorial patterns of space use and inform transmission of pathogens^50,66^ affecting both livestock and humans^67,68^. Ultimately, better understanding patterns of territoriality and space use between animals can inform management and conservation plans aimed at understanding social interactions, disease dynamics, and movement patterns of animals^43^.

## Study areas

Our work was conducted at Archbold’s—Buck Island Ranch (27° 10′ N, 81° 2  l′ W, elevation 6–12 m), located in a biologically diverse area of central Florida^69^. The study area (4250 hectare) contains over 600 ephemeral wetlands and an extensive network of drainage ditches. Common tree species on the ranch include live oak (*Quercus virginiana*) and cabbage palm (*Sabal palmetto*) which are interspersed within extensive areas of bahiagrass (*Paspalum notatum*) and native C4 grasses. Average temperatures range from 26 °C in July to 13 °C in January^70^. Rainfall averages 1365 mm per year with more than 60% of precipitation occurring between June and September. There are two biologically distinct seasons based on temperature and rainfall, a dry (December–April) and wet (June–October) season.

## Methods

### Animal capture

Wild pigs were captured between May 2015 and May 2017 using open-topped box traps (2.5 m deep × 1.25 m wide × 1.5 m high) and corral traps (5 m diameter), both with drop doors. Traps were pre- baited for 10–14 days with soured corn. After capture, adult wild pigs were immobilized using Telazol 50 mg/ml (Zoetis. Parsippany, New Jersey, USA) and Xylazine 100 mg/ml (Akorn Inc. Lake Forest, Illinois, USA ) mixed following the recommendations of established dosages (Telazol, 4.40 mg/kg; Xylazine, 2.5 mg/kg)^71–73^. During anesthesia wild pigs were measured, sexed, physiological samples taken for other studies and fitted with one of three global positioning system (GPS) collars (3300L Lotek Wireless Inc., Newmarket, Ontario, Canada; CatLog Gen 2 GPS, Perthold Engineering LLC, Dallas, Texas, USA 75243; or igot-U, Mobile Action Technology, Inc., Taiwan) that were programmed to record a location every 30 min. Wild pig capture and handling procedures were administered under approved University of Florida Animal Care and Use Committee protocols (#201408495 and #201808495) and all methods were performed in accordance with the relevant guidelines and regulations**.** The study complies with ARRIVE guidelines.

### Space use and animal interactions

We estimated home ranges and core areas using the Brownian bridge movement model^74^ (BBMM) in the package “mkde”^75^ in program R^76^. Home ranges were estimated using a 99.999% cumulative probability and core areas were estimated using an 80% cumulative probability. An 80% cumulative probability was selected so that approximately 25% of the home range was conserved in the core area (following Sawyer and Kauffman^77^; Fig. 2). A minimum of six weeks of GPS data were required for an individual to be included in analyses and if more than one adult female was collared within the same social group, the individual with the greater number of locations was included in analyses. Spatial data were available for 3 wet and 2 dry seasons (Wet 2015, Dry 2016, Wet 2016, Dry 2017, Wet 2017), which we refer to as sampling periods to avoid confusion when wet and dry seasons are combined in analyses.

Overlap in space use was used to evaluate indirect interactions among individuals, which can be highly correlated with direct interactions (physical contact between individuals) and represents the potential for animals to interact^78,79^. Spatial overlap is a useful metric to evaluate potential interactions when GPS data is available at different time periods among animals and when it is unclear what distance between concurrent GPS locations should be used to define an interaction^54,78–80^. We estimated two overlap metrics that measure the strength (space-use overlap) and number (degree) of potential interactions. Space-use overlap among animals was estimated using the utilization distribution overlap index^81^ (UDOI), which is a normalized index ranging from 0, when there is no overlap, to > 1 if non-uniform utilization distributions exhibit a high degree of overlap. Degree measured the number of potential interactions by summing the number of animals an individual spatially overlapped with^82,83^.

For objectives one and two, we quantified the effects of sex and season on wild pig home range size and spatial overlap using generalized linear mixed-effect models regression models^84^ and AIC model selection^85^. We analyzed home range size using a Gamma distribution because home range size was non-normal and right skewed. We accounted for yearly variation and repeated observations of individuals by including year and individual as random effects (Year and ID, Supplementary Table S1a). We analyzed space-use overlap (UDOI) of home ranges for each interacting dyad (pair of individuals), using a log normal distribution. In models, random effects included the dyadic interaction and the year (Dyad and Year, Supplementary Table S1b). Finally, we evaluated degree using a negative binomial distribution because counts were over dispersed. We accounted for repeated measures by specifying individuals as a random effect (ID, Supplementary Table S1c). Because the number of monitored wild pigs varied among sampling periods (Table 2), we also controlled for differences in the maximum number of potential interactions by including sampling period (e.g. Wet 2015) as a random effect (SP, Supplementary Table S1c). For all analyses, fixed effects included sex of the individual or sexes within the dyad [female–female (F–F), female–male (F–M), or male–male (M–M)], season (Wet or Dry), and the interaction of sex and season (Supplementary Table S1). We generated 8 a priori models including an intercept only null model and combinations of random and fixed effects (Supplementary Table S1).

To evaluate patterns of territoriality, we evaluated what factors were important predictors of overlap by comparing home range to home range interactions (HR–HR) with home range to core area interactions (HR–CA), as well as HR–CA interactions with core area to core area (CA–CA) interactions. For each interacting dyad per sampling period, we estimated UDOI values from each interaction level (HR–HR, HR–CA, and CA–CA). Variation in UDOI was evaluated using a log normal distribution. In models, random effects included the dyadic interaction and the year. Fixed effects included the space use level, sexes within the dyad, season, and all two-way and three-way interaction terms (Table 3a,b). We also calculated degree per individual for each space use level and evaluated degree with a negative binomial distribution because counts were overdispersed. Random effects included individual animal and the sampling period. Fixed effects included space use level, sex, season, and all two-way and three-way interaction terms (Table 3c,d). For both UDOI and degree, 15 a priori models were identified that included an intercept-only model (i.e., model without any covariates) and all combinations of level, sex, season, and interaction terms (Table 3). We ranked models using Akaike Information Criterion^86^ and validated each model by graphically examining the residuals and inspecting the QQ-plot. All analyses were conducted in R version 3.5.2^76^ using package “lme4”^84^.

## Supplementary Information

Supplementary Information.

## References

[1] Schoener TW, Schoener A (1982). Intraspecific variation in home-range size in some Anolis lizards. Ecology.

[2] Grigione MM, Beier P, Hopkins RA, Neal D, Padley WD, Schonewald CM, Johnson ML (2002). Ecological and allometric determinants of home-range size for mountain lions (*Puma concolor*). Anim. Conserv..

[3] Wolf JB, Mawdsley D, Trillmich F, James R (2007). Social structure in a colonial mammal: Unravelling hidden structural layers and their foundations by network analysis. Anim. Behav..

[4] Gehrt SD, Frttzell EK (1997). Sexual differences in home ranges of raccoons. J. Mammal..

[5] Clutton-Brock TH, Iason GR, Guinness FE (1987). Sexual segregation and density-related changes in habitat use in male and female Red deer (*Cervus elaphus*). J. Zool..

[6] Ji W, White PC, Clout MN (2005). Contact rates between possums revealed by proximity data loggers. J. Appl. Ecol..

[7] Böhm M, Palphramand KL, Newton-Cross G, Hutchings MR, White PC (2008). Dynamic interactions among badgers: Implications for sociality and disease transmission. J. Anim. Ecol..

[8] Hamede RK, Bashford J, McCallum H, Jones M (2009). Contact networks in a wild Tasmanian devil (*Sarcophilus harrisii*) population: Using social network analysis to reveal seasonal variability in social behaviour and its implications for transmission of devil facial tumour disease. Ecol. Lett..

[9] Ostfeld RS, Glass GE, Keesing F (2005). Spatial epidemiology: An emerging (or re-emerging) discipline. Trends Ecol. Evol..

[10] Mitani JC, Watts DP, Amsler SJ (2010). Lethal intergroup aggression leads to territorial expansion in wild chimpanzees. Curr. Biol..

[11] Cubaynes S, MacNulty DR, Stahler DR, Quimby KA, Smith DW, Coulson T (2014). Density-dependent intraspecific aggression regulates survival in northern Yellowstone wolves (*Canis lupus*). J. Anim. Ecol..

[12] Wittemyer G, Getz WM, Vollrath F, Douglas-Hamilton I (2007). Social dominance, seasonal movements, and spatial segregation in African elephants: A contribution to conservation behavior. Behav. Ecol. Sociobiol..

[13] McGuire JM, Scribner KT, Congdon JD (2013). Spatial aspects of movements, mating patterns, and nest distributions influence gene flow among population subunits of Blanding’s turtles (*Emydoidea blandingii*). Conserv. Genet..

[14] Kurvers RH, Krause J, Croft DP, Wilson AD, Wolf M (2014). The evolutionary and ecological consequences of animal social networks: Emerging issues. Trends Ecol. Evol..

[15] Loveridge AJ, Macdonald DW (2001). Seasonality in spatial organization and dispersal of sympatric jackals (*Canis mesomelas* and *C. adustus*): Implications for rabies management. J. Zool..

[16] Snijders L, Blumstein DT, Stanley CR, Franks DW (2017). Animal social network theory can help wildlife conservation. Trends Ecol. Evol..

[17] Burt WH (1943). Territoriality and home range concepts as applied to mammals. J. Mammal..

[18] Schoener TW (1968). Sizes of feeding territories among birds. Ecology.

[19] Kaufman JH (1983). On the definitions and functions of dominance and territoriality. Biol. Revue.

[20] Maher CR, Lott DF (1995). Definitions of territoriality used in the study of variation in vertebrate spacing systems. Anim. Behav..

[21] Powell RA (2000). Animal home ranges and territories and home range estimators. Res. Tech. Anim. Ecol. Controversies Conseq..

[22] Kerr GD, Bull CM (2006). Exclusive core areas in overlapping ranges of the sleepy lizard, *Tiliqua rugosa*. Behav. Ecol..

[23] DiPierro E, Molinari A, Tosi G, Wauters LA (2008). Exclusive core areas and intrasexual territoriality in Eurasian red squirrels (*Sciurus vulgaris*) revealed by incremental cluster polygon analysis. Ecol. Res..

[24] Poole KG (1995). Spatial organization of a lynx population. Can. J. Zool..

[25] Chamberlain MJ, Leopold BD (2002). Spatio-temporal relationships among adult raccoons (*Procyon lotor*) in central Mississippi. Am. Midl. Nat..

[26] Darden SK, Dabelsteen T (2008). Acoustic territorial signaling in a small, socially monogamous canid. Anim. Behav..

[27] Gabor TM, Hellgren EC, Van Den Bussche RA, Silvy NJ (1999). Demography, sociospatial behaviour and genetics of feral pigs (*Sus scrofa*) in a semi-arid environment. J. Zool..

[28] Seiler N, Boesch C, Mundry R, Stephens C, Robbins MM (2017). Space partitioning in wild, non-territorial mountain gorillas: The impact of food and neighbours. R. Soc. Open Sci..

[29] Podgórski T, Baś G, Jędrzejewska B, Sönnichsen L, Śnieżko S, Jędrzejewski W, Okarma H (2013). Spatiotemporal behavioral plasticity of wild boar (*Sus scrofa*) under contrasting conditions of human pressure: Primeval forest and metropolitan area. J. Mammal..

[30] Podgórski T, Lusseau D, Scandura M, Sonnichsen L, Jedrzejewska B (2014). Long-lasting, kin-directed female interactions in a spatially structured wild boar social network. PLoS One.

[31] Keiter DA, Beasley JC (2017). Hog heaven? Challenges of managing introduced wild pigs in natural areas. Nat. Areas J..

[32] Lewis JS, Farnsworth ML, Burdett CL, Theobald DM, Gray M, Miller RS (2017). Biotic and abiotic factors predicting the global distribution and population density of an invasive large mammal. Sci. Rep..

[33] Singer FJ, Otto DK, Tipton AR, Hable CP (1981). Home ranges, movements, and habitat use of European wild boar in Tennessee. J. Wildl. Manag..

[34] Saunders G, Kay B (1990). Movements of feral pigs at Sunny Corner, New South Wales. Wildl. Res..

[35] Boitani L, Mattei L, Nonis D, Corsi F (1994). Spatial and activity patterns of wild boars in Tuscany, Italy. J. Mammal..

[36] Dexter N (1999). The influence of pasture distribution, temperature and sex on home-range size of feral pigs in a semi-arid environment. Wildl. Res..

[37] Calenge C, Maillard D, Vassant J, Brandt S (2002). Summer and hunting season home ranges of wild boar (*Sus scrofa*) in two habitats in France. Game Wildl. Sci..

[38] Hayes R, Riffell S, Minnis R, Holder B (2009). Survival and habitat use of feral hogs in Mississippi. Southeast. Nat..

[39] Fattebert J, Baubet E, Slotow R, Fischer C (2017). Landscape effects on wild boar home range size under contrasting harvest regimes in a human-dominated agro-ecosystem. Eur. J. Wildl. Res..

[40] Clontz LM, Pepin KM, VerCauteren KC, Beasley JC (2021). Influence of biotic and abiotic factors on home range size and shape of invasive wild pigs (*Sus scrofa*). Pest Manag. Sci..

[41] Mcloughlin PD, Ferguson SH, Messier F (2000). Intraspecific variation in home range overlap with habitat quality: A comparison among brown bear populations. Evol. Ecol..

[42] Golabek KA, Ridley AR, Radford AN (2012). Food availability affects strength of seasonal territorial behaviour in a cooperatively breeding bird. Anim. Behav..

[43] Kilgo JC, Garabedian JE, Vukovich M, Schlichting PE, Byrne ME, Beasley JC (2021). Food resources affect territoriality of invasive wild pig sounders with implications for control. Sci. Rep..

[44] Geist V (1977). A comparison of social adaptations in relations to ecology in gallinaceous bird and ungulate societies. Annu. Rev. Ecol. Syst..

[45] Ilse LM, Hellgren EC (1995). Resource partitioning in sympatric populations of collared peccaries and feral hogs in southern Texas. J. Mammal..

[46] Sparklin BD, Mitchell MS, Hanson LB, Jolley DB, Ditchkoff SS (2009). Territoriality of feral pigs in a highly persecuted population on Fort Benning, Georgia. J. Wildl. Manag..

[47] Barrett R (1978). The feral hog at Dye Creek ranch, California. Hilgardia.

[48] Baber DW, Coblentz BE (1986). Density, home range, habitat use, and reproduction in feral pigs on Santa Catalina Island. J. Mammal..

[49] Kay SL, Fischer JW, Monaghan AJ, Beasley JC, Boughton R, Campbell TA, Cooper SM, Ditchkoff SS, Hartley SB, Kilgo JC, Wisely SM (2017). Quantifying drivers of wild pig movement across multiple spatial and temporal scales. Mov. Ecol..

[50] Pepin KM (2016). Contact heterogeneities in feral swine: implications for disease management and future research. Ecosphere.

[51] Singh JS, Yadava PS (1974). Seasonal variation in composition, plant biomass, and net primary productivity of a tropical grassland at Kurukshetra, India. Ecol. Monogr..

[52] Swemmer AM, Knapp AK, Snyman HA (2007). Intra-seasonal precipitation patterns and above-ground productivity in three perennial grasslands. J. Ecol..

[53] Harless ML, Walde AD, Delaney DK, Pater LL, Hayes WK (2009). Home range, spatial overlap, and burrow use of the desert tortoise in the West Mojave Desert. Copeia.

[54] Lewis JS, Logan KA, Alldredge MW, Theobald DM, VandeWoude S, Crooks KR (2017). Contact networks reveal potential for interspecific interactions of sympatric wild felids driven by space use. Ecosphere.

[55] Weber N, Carter SP, Dall SRX, Delahay RJ, McDonald JL, Bearhop S, McDonald RA (2013). Badger social networks correlate with tuberculosis infection. Curr. Biol..

[56] Vander Waal KL, Obanda V, Omondi GP, McCowan B, Wang H, Fushing H, Isbell LA (2016). The “strength of weak ties” and helminth parasitism in giraffe social networks. Behav. Ecol..

[57] Podgórski T, Apollonio M, Keuling O (2018). Contact rates in wild boar populations: Implications for disease transmission. J. Wildl. Manag..

[58] D’Andrea L, Durio P, Perrone A, Pirone S (2014). Preliminary data of the wild boar (*Sus scrofa*) space use in mountain environment. IBEX J. Mountain Ecol..

[59] Keuling O, Stier N, Roth M (2008). Annual and seasonal space use of different age classes of female wild boar *Sus scrofa* L. Eur. J. Wildl. Res..

[60] Hixon MA (1980). Food production and competitor density as the determinants of feeding territory size. Am. Nat..

[61] Bastille-Rousseau G, Schlichting PE, Keiter DA, Smith JB, Kilgo JC, Wittemyer G, Vercauteren KC, Beasley JC, Pepin KM (2021). Multi-level movement response of invasive wild pigs (*Sus scrofa*) to removal. Pest Manag. Sci..

[62] Maher CR, Lott DF (2000). A review of ecological determinants of territoriality within vertebrate species. Am. Midl. Nat..

[63] Mendl M, Randle K, Pope S (2002). Young female pigs can discriminate individual differences in odours from conspecific urine. Anim. Behav..

[64] Marsh MK, Hutchings MR, McLeod SR, White PCL (2011). Spatial and temporal heterogeneities in the contact behaviour of rabbits. Behav. Ecol. Sociobiol..

[65] Yang A, Schlichting PE, Wight B, Anderson WM, Chinn SM, Wilber MQ, Miller RS, Beasley JC, Boughton RK, VerCauteren KC, Wittemyer G (2021). Effects of social structure and management on risk of disease establishment in wild pigs. J. Anim. Ecol..

[66] Lavelle MJ, Fischer JW, Phillips GE, Hildreth AM, Campbell TA, Hewitt DG, Hygnstrom SE, Vercauteren KC (2014). Assessing risk of disease transmission: Direct implications for an indirect science. Bioscience.

[67] Gortázar C, Ferroglio E, Hofle U, Frolich K, Vicente J (2007). Diseases shared between wildlife and livestock: A European perspective. Eur. J. Wildl. Res..

[68] Miller RS, Sweeney SJ, Slootmaker C, Grear DA, Salvo PA, Kiser D, Shwiff SA (2017). Cross-species transmission potential between wild pigs, livestock, poultry, wildlife, and humans: Implications for disease risk management in North America. Sci. Rep..

[69] Abrahamson WG, Johnson AF, Layne JN, Peroni PA (1984). Vegetation of the Archbold Biological Station, Florida: An example of the southern Lake Wales ridge. Florida Sci..

[70] Boughton EH, Boughton RK (2014). Modification by an invasive ecosystem engineer shifts a wet prairie to a monotypic stand. Biol. Invasions.

[71] Ko J, Williams B, Smith V, McGrath C, Jacobson J (1993). Comparison of Telazol, Telazol–ketamine, Telazol–xylazine, and Telazol–ketamine–xylazine as chemical restraint and anesthetic induction combination in swine. Lab Anim. Sci..

[72] Gabor TM, Hellgren EC, Silvy NJ (1997). Immobilization of collared peccaries (*Tayassu tajacu*) and feral hogs (*Sus scrofa*) with Telazol® and xylazine. J. Wildl. Dis..

[73] Sweitzer RA, Ghneim GS, Gardner IA, Vuren DV, Gonzales BJ, Boyce WM (1997). Immobilization and physiological parameters associated with chemical restraint of wild pigs with Telazol® and xylazine hydrochloride. J. Wildl. Dis..

[74] Horne JS, Garton EO, Krone SM, Lewis JS (2007). Analyzing animal movements using Brownian bridges. Ecology.

[75] Tracey, J. A. mkde. R Core Development Team. (2014). https://cran.r-project.org/web/packages/mkde/index.Html. Accessed 27 Mar 2021

[76] R Development Core Team. R: a language and environment for statistical computing, version 3.5.1. R Foundation for Statistical Computing, Vienna, Austria. (2018). https://www.r-project.org/. Accessed 27 Mar 2021

[77] Sawyer H, Kauffman MJ (2011). Stopover ecology of a migratory ungulate. J. Anim. Ecol..

[78] Vander Wal E, Laforge MP, McLoughlin PD (2014). Density dependence in social behaviour: Home range overlap and density interacts to affect conspecific encounter rates in a gregarious ungulate. Behav. Ecol. Sociobiol..

[79] Schauber EM, Nielsen CK, Kjær LJ, Anderson CW, Storm DJ (2015). Social affiliation and contact patterns among white-tailed deer in disparate landscapes: Implications for disease transmission. J. Mammal..

[80] Robert K, Garant D, Pelletier F (2012). Keep in touch: Does spatial overlap correlate with contact rate frequency?. J. Wildl. Manag..

[81] Fieberg J, Kochanny CO (2005). Quantifying home-range overlap: The importance of the utilization distribution. J. Wildl. Manag..

[82] Newman ME (2003). The structure and function of complex networks. SIAM Rev..

[83] Wey T, Blumstein DT, Shen W, Jordan F (2008). Social network analysis of animal behaviour: A promising tool for the study of sociality. Anim. Behav..

[84] Bates, D., Maechler, M., Bolker, B., & Walker, S. lme4: linear mixed effects models using Eigen and S4. R package version 1.1-9. (2014) https://cran.rproject.org/package/lme4. (accessed 30 Jan 2019).

[85] Burnham KP, Anderson DR (2002). A Practical Information-Theoretic Approach. Model Selection and Multi-model Inference.

[86] Akaike, H. Information theory and an extension of the maximum likelihood principle. In *Second international symposium on information theory*. (eds. Petrov, B. N. & Csaki, F.) 267–281 (Academiai Kiado, 1973).

