# Supplementary material for: Seasonal variation in space use and territoriality in a large mammal (Sus scrofa)
Source: Sci Rep. 2022 Mar 7;12:4023. doi: 10.1038/s41598-022-07297-y (PMC8901613; doi:10.1038/s41598-022-07297-y)
Supplement: Supplementary file 1 — Supplementary Information. [file 41598_2022_7297_MOESM1_ESM.docx]

Table S1. Results of generalized mixed-effects linear-regression models evaluating variation in home range size and spatial overlap of wild pigs at Buck Island Ranch, FL. Home range size was estimated via the Brownian bridge movement model using a 99.999% cumulative probability. Spatial overlap was measured by utilization distribution of overlap index (UDOI, b.) and degree (c.). Random effects include the two individuals that are interacting (Dyad) or the individual (ID) and the year (Year) or the sampling period (e.g. Wet 2015, SP). Models include sex (Sex), season, and an interaction term as fixed effects. For UDOI, sex indicates the sex of the two interacting individuals (two females, female and male, or two males). For degree, sex is the sex of the individual used to determine degree. Model output includes the number of parameters (K), AIC values, ΔAIC values, AIC weights (wi), and residual deviance (Dev.).

| a. Home Range Size | K | AIC | ΔAIC | *w_i_* | Dev. |
| --- | --- | --- | --- | --- | --- |
| (1\|ID) + Sex | 4 | 340.90 | 0.00 | 0.95 | -166.19 |
| (1\|ID) + Sex + Season | 8 | 347.16 | 6.25 | 0.04 | -164.57 |
| (1\|ID) | 3 | 351.50 | 10.60 | 0.00 | -172.59 |
| (1\|ID) + (1\|Year) | 4 | 353.72 | 12.82 | 0.00 | -172.59 |
| (1\|ID) + Sex + Season + (Sex × Season) | 12 | 355.10 | 14.20 | 0.00 | -163.22 |
| (1\|ID) + Season | 7 | 356.46 | 15.55 | 0.00 | -170.45 |
| Intercept-only | 2 | 446.09 | 0.00 | 0.00 | -220.97 |
| (1\|Year) | 3 | 450.25 | 109.35 | 0.00 | -221.97 |
|  |  |  |  |  |  |
| b. Space-Use Overlap | K | AIC | ΔAIC | *w_i_* | Dev. |
| (1\|Dyad) + Season | 4 | -670.08 | 0.00 | 0.58 | 339.09 |
| (1\|Dyad) + Sex + Season | 6 | -667.88 | 2.20 | 0.19 | 340.03 |
| (1\|Dyad) | 3 | -666.54 | 3.54 | 0.10 | 336.30 |
| (1\|Dyad) + Sex | 5 | -665.88 | 4.21 | 0.07 | 338.00 |
| (1\|Dyad) + (1\|Year) | 4 | -664.51 | 5.58 | 0.04 | 336.30 |
| (1\|Dyad) + Sex + Season + (Sex × Season) | 8 | -664.11 | 5.98 | 0.03 | 340.21 |
| Intercept-only | 2 | -635.19 | 34.89 | 0.00 | 319.61 |
| (1\|Year) | 3 | -633.16 | 36.93 | 0.00 | 319.61 |
|  |  |  |  |  |  |
| c. Degree: HRHR-HRCA | K | AIC | ΔAIC | *w_i_* | Dev. |
| (1\|ID) + (1\|SP) + Sex | 5 | 377.95 | 0.00 | 0.61 | -183.57 |
| (1\|ID) + (1\|SP) + Sex + Season | 6 | 379.75 | 1.80 | 0.25 | -183.29 |
| (1\|ID) + (1\|SP) + Sex + Season + (Sex × Season) | 7 | 381.02 | 3.06 | 0.13 | -182.72 |
| (1\|ID) + (1\|SP) | 4 | 388.14 | 10.19 | 0.00 | -189.80 |
| (1\|ID) + (1\|SP) + Season | 5 | 389.76 | 11.81 | 0.00 | -189.47 |
| (1\|SP) | 3 | 393.38 | 15.42 | 0.00 | -193.53 |
| (1\|ID) | 3 | 407.99 | 30.03 | 0.00 | -200.83 |
| Intercept-only | 2 | 416.46 | 38.51 | 0.00 | -206.15 |


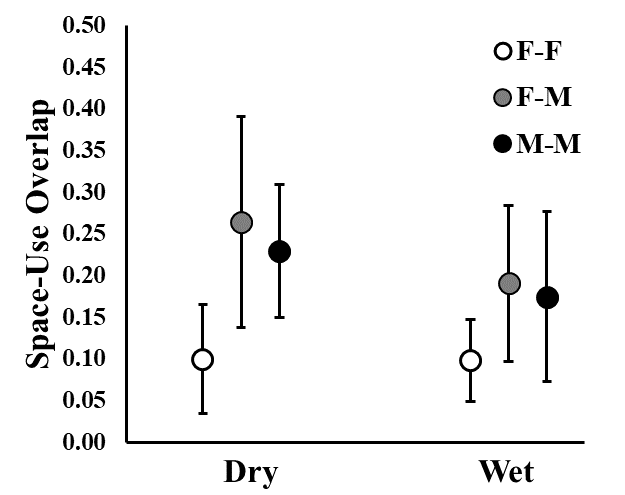


Figure S1. Average space-use overlap estimates (measured by utilization distribution of overlap index (UDOI) with associated 95% confidence intervals for wild pigs at Buck Island Ranch, FL in the dry (Dry) and wet (Wet) season. Space-use overlap estimates were created for home range overlap of female-female (F-F), female-male (F-M), and male-male (M-M) interactions.

1. Wet 2015 b. Dry 2016


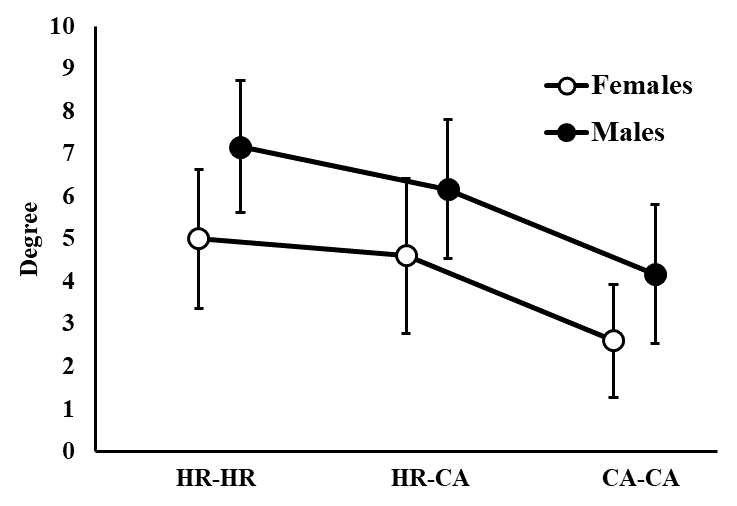

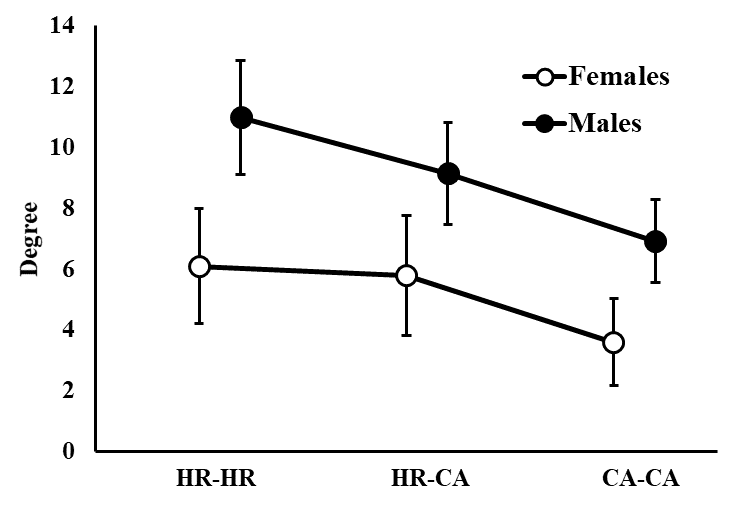


c. Wet 2016 d. Dry 2017


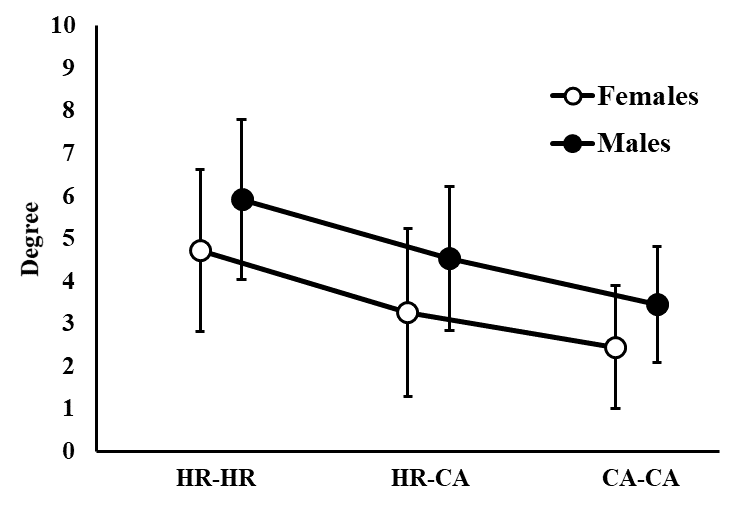

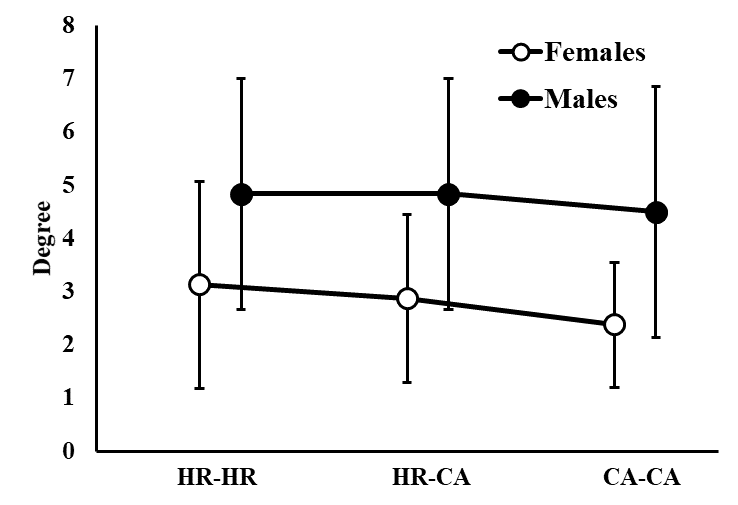


e. Wet 2017


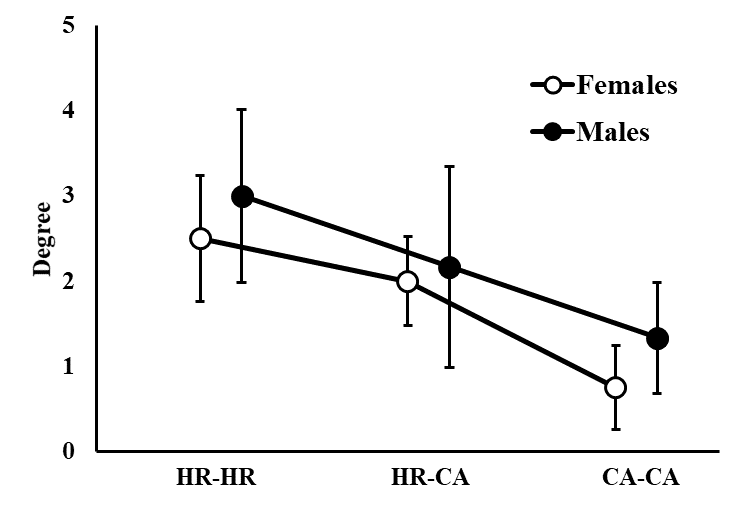


Figure S2. Average degree with associated 95% confidence intervals for wild pigs at Buck Island Ranch, FL in the Wet 2015 (a.), Dry 2016 (b.), Wet 2016 (c.), Dry 2017 (d.), and Wet 2017 (e.) seasons. Estimates were created for males and females for home range to home range (HR-HR), home range to core area (HR-CA) and core area to core area (CA-CA) interactions. Note the varying scale on the y-axis.
